# Generation of functional HLA-DR*1101 tetramers receptive for loading with pathogen or tumour derived synthetic peptides

**DOI:** 10.1186/1471-2172-6-24

**Published:** 2005-12-05

**Authors:** Monica Moro, Virginia Cecconi, Chiara Martinoli, Eliana Dallegno, Barbara Giabbai, Massimo Degano, Nicholas Glaichenhaus, Maria Pia Protti, Paolo Dellabona, Giulia Casorati

**Affiliations:** 1 Experimental Immunology Unit, Cancer Immunotherapy and Gene Therapy Program, Dept. of Oncology, DIBIT San Raffaele Scientific Institute, 20132 Milano, Italy; 2 Biocrystallography Unit, DIBIT San Raffaele Scientific Institute, 20132 Milano, Italy; 3 INSERM E0344, Universite de Nice-Sophia Antipolis, Valbonne, France; 4 Tumour Immunology Unit, Cancer Immunotherapy and Gene Therapy Program, Dept. of Oncology, DIBIT San Raffaele Scientific Institute, 20132 Milano, Italy

## Abstract

**Background:**

MHC class I-peptide tetramers are currently utilised to characterize CD8^+ ^T cell responses at single cell level. The generation and use of MHC class II tetramers to study antigen-specific CD4^+ ^T cells appears less straightforward. Most MHC class II tetramers are produced with a homogeneously built-in peptide, reducing greatly their flexibility of use. We attempted the generation of "empty" functional HLA-DR*1101 tetramers, receptive for loading with synthetic peptides by incubation. No such reagent is in fact available for this HLA-DR allele, one of the most frequent in the Caucasian population.

**Results:**

We compared soluble MHC class II-immunoglobulin fusion proteins (HLA-DR*1101-Ig) with soluble MHC class II protein fused with an optimised Bir site for enzymatic biotynilation (HLA-DR*1101-Bir), both produced in insect cells. The molecules were multimerised by binding fluorochrome-protein A or fluorochrome-streptavidin, respectively. We find that HLA-DR*1101-Bir molecules are superior to the HLA-DR*1101-Ig ones both in biochemical and functional terms. HLA-DR*1101-Bir molecules can be pulsed with at least three different promiscuous peptide epitopes, derived from Tetanus Toxoid, influenza HA and the tumour associated antigen MAGE-3 respectively, to stain specific CD4^+ ^T cells. Both staining temperature and activation state of CD4^+ ^T cells are critical for the binding of peptide-pulsed HLA-DR*1101-Bir to the cognate TCR.

**Conclusion:**

It is therefore possible to generate a soluble recombinant HLA-DR*1101 backbone that is receptive for loading with different peptides to stain specific CD4^+ ^T cells. As shown for other HLA-DR alleles, we confirm that not all the strategies to produce soluble HLA-DR*1101 multimers are equivalent.

## Background

The direct *ex-vivo *visualization and quantification of antigen-specific T cells is key to the characterisation of complex immune responses. The antigen specificity of T cells is determined by the highly specific interaction between their TCR and the cognate MHC-peptide complex. The low affinity and fast off-rate of this interaction, however, precludes its exploitation to identify specific T cells [1-5]. Tetramer technology allows to circumvent this limitation, since the overall increased avidity of the MHC multimers compensates for the low affinity of the TCR-peptide/MHC interaction [6,7]. MHC class I tetramers are now indeed largely used to characterise CD8^+ ^T cells in many basic or clinical settings [8-13]. By contrast, the generation of functional soluble peptide/MHC class II multimers, to characterise CD4^+ ^T cell responses, seems somewhat less straightforward. Structural differences between MHC class I and class II molecules might account for the greater difficulties to standardise the generation and production of MHC class II tetramers. In MHC class I molecules, the α chain alone contains the complete peptide-binding groove and it is stable in the soluble form when associated with the β_2_-microglobulin chain [14]. In contrast, both the α and β chain of the MHC class II molecules contributes to the formation of the peptide-binding site, and molecular "zippers" are necessary to guide the correct inter-chain dimerisation to stabilize the soluble recombinant αβ heterodimers. Moreover, owing to the different structure of the peptide-binding groove, MHC class I and II molecules bind peptides stably and in an homogeneous frame or unstably and in heterogeneous frames, respectively [15,16]. A variety of approaches have been used to overcome these hurdles and, in general, it appears that there does not exist a universal way to produce soluble MHC class II molecules. Most of the adopted strategies, however, generate MHC-class II molecules endowed with little or no flexibility in terms of peptide loading. For instance, HLA-DR1 and H2-IE^k ^have been successfully produced in bacteria [17-19]. In analogy with the production of soluble MHC class I molecules, their α and β chains are separately produced in *E. coli *as denatured proteins, which are allowed to refold correctly as αβ heterodimer in the presence of a single peptide. Once refolded, however, the MHC class II heterodimer cannot exchange the peptide any longer. Another way to produce functional class II tetramers relies on the generation of soluble MHC class II αβ heterodimers engineered with a covalently linked peptide at the N-terminus of either chain, via a flexible amino acid linker of various lengths. This approach results in a homogeneous binding of the target peptides to the soluble MHC class II heterodimer [20-23]. This strategy has been successfully applied to both human and mouse MHC class II molecules produced in the prokaryotic as well as the eukaryotic expression system. The latter system is generally based on insect or mammalian cells, which offer the advantage over bacteria of producing proteins in native conformation and in glycosylated form. Although the presence of a covalently linked peptide improves the yield of functional soluble MHC class II tetramers, it negatively impacts on their flexibility of use, forcing the separate production of a soluble MHC II molecule for each peptide of interest.

The generation of soluble recombinant MHC class II molecules without covalently linked peptide has been successfully used with HLA-DR*0401 and HLA-DR0*404 molecules [23-26]. This approach permits an increased flexibility in the usage of recombinant HLA-DR monomers, which are loaded with the desired synthetic peptides by *in vitro *incubation.

Moreover, no univocal strategy to multimerise soluble recombinant MHC class II monomers has been yet devised. For this purpose, in fact, two main modifications are currently introduced at the C-terminus of the MHC class II monomer. In the first approach, the extracellular domains of either MHC class II α or β chain are linked to the Ig constant region. Chimeric MHC-class II-Ig molecules are generally multimerised by using protein A, which is bivalent, thus generating a tetrameric (dimer of dimers) peptide/MHC complex [21,22,27,28]. Alternatively, the extracellular domains of MHC class II α or β chain are linked to an enzymatic biotinylation site (Bir) [19,20,24,29]. After labelling with biotin, tetramerisation of MHC class II-Bir monomers is accomplished by coupling with fluorochrome-conjugate streptavidin [19,20,24].

We were interested in generating HLA-DR*1101 tetramers to characterise CD4^+ ^T cell responses to several peptide epitopes derived from either environmental or tumour-associated antigens. This molecule represents one of the most frequent HLA-DR allele in Caucasians, being expressed in up to 20% of the population, we therefore decided to attempt the production of HLA-DR*1101 heterodimers without covalently linked built-in peptide, receptive for loading the desired peptide by incubation. Since no information is currently available on functional empty tetramers made of this HLA-DR allele, and given the premises outlined above, we compared two strategies for the production of soluble HLA-DR*1101 molecules in insect cells: the first generating soluble chimeric HLA-DR*1101-Ig molecules, the second HLA-DR*1101-Bir ones. We show here that it is indeed possible to produce functional HLA-DR*1101 tetramers, which can be loaded with the desired peptide after the purification of the protein. For this purpose, HLA-DR*1101-Bir molecules resulted superior to the HLA-DR*1101-Ig chimeric constructs.

## Results

### Production and biochemical characterisation of soluble HLA-DR*1101 molecules

**Figure 1:**
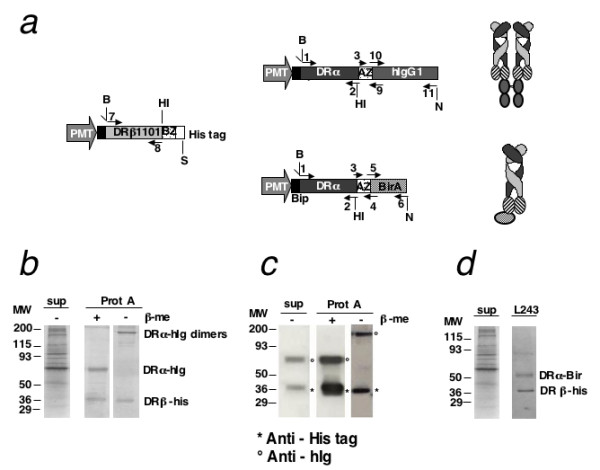
Construction of soluble recombinant HLA-DR*1101-Ig and HLA-DR*1101-Bir molecules. Structure and homogeneity of purified soluble HLA-DR*1101 recombinant molecules. (a) Schematic representation of HLA-DR*1101-Ig and HLA-DR*1101-Bir constructs. The extracellular region of HLA-DR*1101β is fused with the Basic Zipper (BZ) and His tag. The HLA-DRα chain is fused with the Acid Zipper (AZ) and the human (h)IgG1 constant region or the biotynilation site BirA, respectively. All constructs are cloned into the pMT/V5/His *Drosophila *expression vector, in frame with the *Drosophila *leader sequence Bip, under the control of a *metallotioneine *promoter, inducible by CuSO4 addition. The HLA-DR*1101-Ig molecule is secreted into the supernatant of *Drosophila *cells as dimer of HLA-DR*1101α/β heterodimers, because of a disulphide bond between two hIgG domains. (b) SDS-PAGE of HLA-DR*1101-Ig molecules secreted into the supernatant of S2 cells (sup) after CuSO4 induction, and after purification by protein A affinity chromatography (Prot A). Gels were run either in reducing (+βm) or non-reducing (-βm) conditions. Indicated are the bands corresponding to the migration of the HLA-DRα-hIg homodimers, HLA-DRα-hIg monomers and HLA-DR*1101β-His tag proteins. (c) Western blotting analysis of HLA-DR*1101-Ig molecules, separated on SDS-PAGE as shown in (b). The blotted filter was probed at the same time with the anti-His tag and anti-hIg probes, as indicated in figure. Empty circle and asterisk indicate the migration of the HLA-DRα-hIg and HLA-DR*1101β-His tag, respectively. (d) SDS-PAGE analysis of the HLA-DR*1101-Bir molecule contained into the supernatant (sup) of S2 cells after CuSO4 induction, and after purification by immunoaffinity chromatography with L243 mAb (L243).

To produce HLA-DR*1101-Ig and HLA-DR*1101-Bir molecules, the cDNAs coding the extracellular domains of the HLA-DR α and the HLA-DR*1101 β chain were fused in frame at the 5' end with the sequence encoding for the Drosophila leader peptide Bip, and at the 3' end with the acidic (AZ) and basic (BZ) leucin zipper sequences, respectively (Figure 1a). A sequence coding a hexahistidine tag (His tag) was subsequently added at the 3' end of the HLA-DR*1101 β chain cDNA (Figure 1a).

To generate a chimeric HLA-DR*1101-Ig molecules, the DR α chain was linked at the 3' end with a cDNA encoding for the hinge and Fc fragment of human IgG1 (Figure 1a). To allow targeted biotin-labelling, the HLA-DRα-AZ chain was fused at the 3' end to a sequence coding a peptide recognized by the BirA enzyme (Figure 1a).

Each construct was stably transfected into S2 *Drosophila melanogaster *cells, that were initially grown in selective medium as bulk culture. To stabilise and optimise the production of both recombinant soluble HLA-DR*1101 molecules, bulk cultures of transfected S2 cells were cloned by limiting dilution, and clones producing highest quantity of soluble molecules were selected and expanded.

The HLA-DR*1101-Ig recombinant molecule was secreted in the culture supernatant as dimers of αβ heterodimers, owing to the presence of an inter-chain disulfide bond between two Ig hinge regions, and was purified using an affinity chromatography with protein A-sepharose (Figure 1b). The formation of the disulfide bond between two chimeric HLA-DRα-Ig molecules was confirmed by SDS-PAGE of the purified molecules under reducing and non-reducing condition, followed by western blot analysis using anti-His tag and anti-Ig antisera (Figure 1c).

The HLA-DR*1101-Bir recombinant molecule was secreted as αβ heterodimer by S2 cells and purified by immunoaffinity chromatography with the human HLA-DRα chain-specific mAb L243 (Figure 1d). Noteworthy, the L243 mAb binds a conformational epitope on HLA-DRα which depends on the correct folding of the αβ heterodimer [30,31].

**Figure 2:**
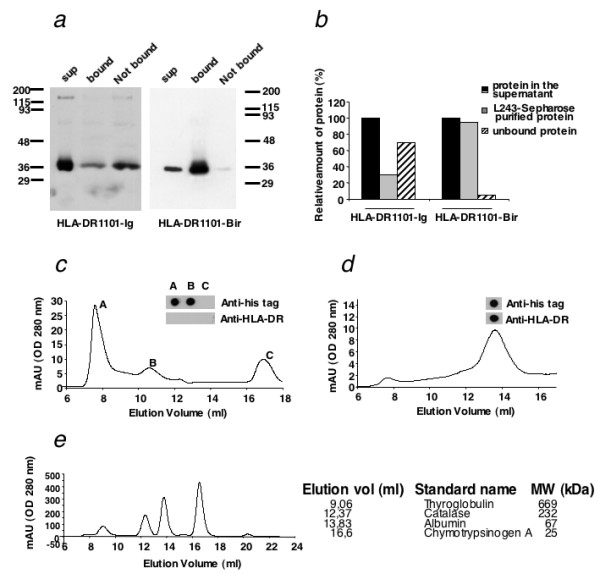
Soluble recombinant HLA-DR*1101-Ig and HLA-DR*1101-Bir molecules display different biochemical properties. Characterization of the native structure and stability of both soluble recombinant HLA-DR*1101 molecules. (a) Immunoprecipitation of HLA-DR*1101-Ig and HLA-DR*1101-Bir molecule with 30 μl of L243-sepharose beads from 1 ml of S2 cells culture supernatant after CuSO4 induction. The lanes contain the following material: 1. sup, 30 μl of culture supernatant before immunoprecipitation; 2. bound, the immunoprecipitated soluble recombinant HLA-DR*1101; and 3. not bound, 30 μl of culture supernatant after immunoprecipitation. Proteins were separated on SDS-page, transferred to filter and revealed with anti his-tag antibody. (b) Densitometric analysis of the protein bands displayed in panel (a), showing the elative efficiency of immunoprecipitation of the two soluble recombinant HLA-DR*1101 proteins with L243-Sepharose beads, as an indirect indicator of the percentage of correctly folded molecule. (c) Size exclusion chromatography of HLA-DR*1101-Ig molecules, after purification of ProtA affinity chromatography. The elution profile of the molecule from a Superdex 200 gel filtration column is shown. Inset shows the dot-blot analysis performed on the protein contained in the indicated elution peaks. Spotted proteins are probed with either anti-His tag antibody, to verify the presence of the HLA-DR*1101 molecule, or L243 mAb (Anti-HLA-DR) to verify the correct conformation of the molecule. (d) Elution profile from Superdex 200 gel filtration column and dot-blot analysis on eluted peaks of HLA-DR11-Bir molecules, performed as described in (c). (e) Calibration profile of the Superdex 200 gel filtration column.

The immunoprecipitation of HLA-DR*1101-Ig and HLA-DR*1101-Bir molecules with the mAb L243 shows that only a little fraction of HLA-DR*1101-Ig molecule can be bound by the mAb, and most of the protein remains in the culture supernatant, suggesting that the secreted HLA-DR*1101-Ig molecules have lost the native conformation recognised by L243 (Figure 2a). A second immunoprecipitation of HLA-DR*1101-Ig molecules from the same supernatant with fresh L243-conjugated beads did not precipitated additional soluble molecule, ruling out that the little protein precipitated was the result of insufficient amounts of mAb-conjugated beads (data not shown). By contrast, as shown in Figure 2a, almost all the HLA-DR*1101-Bir molecule contained in the culture supernatant was immuno-precipitated by the L243-conjugated beads, suggesting that this type of soluble recombinant protein exhibit a correct conformation for mAb binding. Based on the densitometric analysis of the immunoprecipitated molecules, we assumed that only 30% of the HLA-DR*1101-Ig molecule present in the supernatant displayed a correct conformation, in contrast with nearly 100% of correct conformation displayed by the HLA-DR*1101-Bir molecule (Figure 2b). Considering that we have selected transfected S2 cell clones that secreted similar amounts of either soluble recombinant HLA-DR*1101 molecule (not shown), we would need to produce three times more supernatant of HLA-DR*1101-Ig than HLA-DR*1101-Bir to purify the same amount of correctly folded molecule.

To further verify the biochemical properties and stability of the two types HLA-DR*1101 molecules, we performed analytical size-exclusion chromatography on the purified proteins. Figure 2c–d shows the elution profile of HLA-DR*1101-Ig and of HLA-DR*1101-Bir molecules, respectively. The HLA-DR*1101-Ig molecule was eluted in two peaks at elution volume of 7,5 ml, corresponding to the void volume of the column, and 10,6 ml, corresponding to >900 and 500 Kda respectively. Since the expected molecular weight for the HLA-DR*1101-Ig molecule is about 300 KDa, this result suggested a high propensity of the molecule towards aggregation. Moreover, a dot blot analysis could detect the DR1101 protein in the two main elution peaks only when an anti His-tag antibody, but not when the L243 antibody, was utilised. These results further indicated that the HLA-DR*1101-Ig molecule was unstable and tended to progressively aggregate after purification. On the contrary, the elution profile of HLA-DR*1101-Bir (Figure 2d) showed a single symmetric peak at an elution volume of 13,6 ml, corresponding to the expected molecular weight of 80 KDa of the HLA-DR*1101-Bir αβ heterodimer. In this case, the molecule could be detected in a dot-blot with both the anti His-tag mAb and the L243 antibody, indicating that the molecule was correctly folded.

Altogether these data indicate that the HLA-DR*1101-Bir exhibited superior biochemical quality to HLA-DR*1101-Ig molecule both in terms of tertiary/quaternary structure and stability.

### Peptide loading of soluble HLA-DR11 molecules

**Figure 3:**
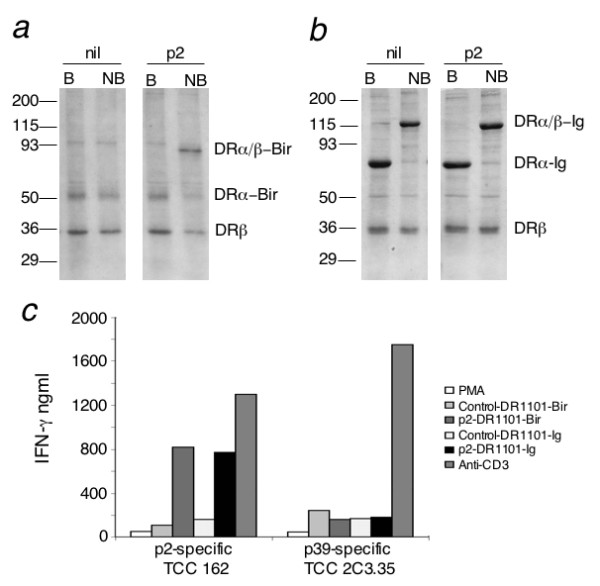
HLA-DR*1101-Ig and HLA-DR*1101-Bir molecules can be loaded with synthetic peptides. Purified soluble HLA-DR*1101-Ig and HLA-DR*1101-Bir molecules were loaded with equal amount of p2 peptide. Stability of the peptide-loaded HLA-DR*1101 αβ heterodimers was determined by running complexes in reducing condition on SDS gels without boiling, or by testing their capacity to elicit IFN-γ production in specific CD4^+ ^T cell clones. (a) SDS-resistance assay of HLA-DR*1101-Bir molecules. Indicated are the protein bands corresponding to the migration of either the HLA-DR*1101-Bir αβ heterodimer, or the single HLA-DRα-Bir or HLA-DR*1101β chains. Lane nil: unloaded HLA-DR*1101-Bir molecules. Lane p2: p2-loaded HLA-DR*1101-Bir molecules. (b) SDS-resistance assay of HLA-DR*1101-Ig molecules. Indicated are the protein bands corresponding to the migration of either the HLA-DR*1101-Ig αβ heterodimer, or the single HLA-DRα-Ig or HLA-DR*1101β chains. Lane nil and p2 are the same as per HLA-DR*1101-bir. (c) IFN-γ production by the p2-specific CD4^+ ^T cell clone (TCC) 162 in response to p2-loaded HLA-DR*1101-Bir or HLA-DR*1101-Ig molecules, attached to plastic. The response of the MAGE-3 p39-specific CD4^+ ^TCC 2C3.35 is shown as control. T cells were cultured in the presence of the indicated peptide-pulsed HLA-DR*1101 recombinant molecules, in the presence of a costimulatory dose of PMA. As control, T cells were cultured in the presence only of sub-optimal doses of PMA (PMA), or activated by plastic-bound anti-CD3 mAb (Anti-CD3). The release of IFN-γ in the culture supernatant was determined by ELISA 48 h later.

The relative disadvantage in the production of HLA-DR*1101-Ig molecules could be compensated by possible peculiar physical properties displayed by this molecule in comparison with HLA-DR*1101-Bir molecules. We speculated in fact that, once pulsed with peptide and tetramerised, the presence of the flexible Ig hinge region in the HLA-DR*1101-Ig construct could represent an advantage and facilitate the binding of cognate TCRs, in comparison with the more rigid HLA-DR*1101-Bir molecules. For this reason, we investigated the functional properties of both HLA-DR*1101-Ig and HLA-DR*1101-Bir molecules. The purified soluble HLA-DR*1101 molecules were loaded with the Tetanus Toxoid-derived peptide TT_830–845 _(p2), a very well characterized promiscuous peptide binding with high affinity to different HLA-DR alleles [32]. Both purified HLA-DR*1101-Ig and HLA-DR*1101-Bir molecules were incubated for 72 hours at 37°C in a mild acidic buffer (pH 5) in the presence of a 50 fold molar excess of p2 peptide. The efficacy of peptide loading was then evaluated by a SDS-resistance assay [33]. The high affinity binding of a peptide to the HLA-DR groove stabilize in fact the association between the HLA-DR α and β chains, making in some cases the αβ heterodimer resistant to the denaturing action of SDS at r.t. [33]. As shown in Figure 3a, the "empty" (nil) HLA-DR*1101-Bir molecules were denatured upon incubation in SDS-containing buffer at r.t., and the DRα and β chains migrated as separated chains in both boiled (B) and not boiled (NB) samples. On the contrary, the binding of p2 substantially stabilised the HLA-DR αβ heterodimer, leading to the appearance of an 80 KDa band in the gel, corresponding to the peptide-MHC complex. Unlike HLA-DR*1101-Bir molecules, the incubation in SDS at r.t. did not induce dissociation of HLA-DR*1101-Ig α and β chains even when the p2 peptide was not added (Figure 3b). These results indicated that the HLA-DR*1101-Ig molecule displayed a stronger interaction between the α and β chains, as compared to HLA-DR*1101-Bir molecules, precluding the quantification of peptide loading by this method.

To characterise further the loading of both soluble recombinant HLA-DR*1101 molecules with the exogenous peptides, we relied on an indirect functional assay, in which p2-loaded HLA-DR*1101 molecules were utilised *in vitro *to activate p2-specific CD4^+ ^T cells. Since the assay was performed in the absence of antigen presenting cells, a sub-optimal dose of phorbol myristate acetate (PMA) was added as costimulatory signal for T cells. As shown in Figure 3c, both TT_830–845_-loaded HLA-DR*1101-Ig and HLA-DR*1101-Bir molecules were able to induce IFN-γ production by p2-specific CD4^+ ^T cells, but not by CD4^+ ^T cells specific for an irrelevant peptide or restricted for another HLA-DR allele. These results indicated that both HLA-DR*1101-Ig and HLA-DR*1101-Bir molecules could be loaded *in vitro *with a peptide of interest.

### Staining of specific CD4^+ ^T cells with peptide-loaded HLA-DR*1101 tetramers

**Figure 4:**
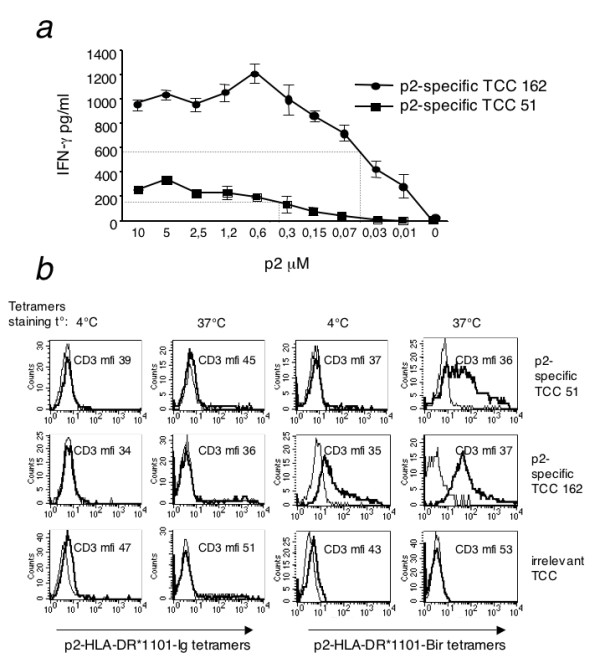
HLA-DR*1101-Bir, but not HLA-DR*1101-Ig, stain specific CD4^+ ^T cells, and display a temperature-dependence. CD4^+ ^TCC specific for p2, endowed with different affinity of recognition, and an irrelevant CD4^+ ^TCC were stained with p2-loaded HLA-DR*1101-Bir tetramers at different temperature. (a) The relative affinity for the p2-HLA-DR*1101 displayed by the two TCC clones 162 and 51 is determined in an IFN-γ-releasing assay following production in response to different doses of p2 peptide. 10^4 ^T cells are incubated with 5 × 10^4 ^HLA-DR*1101 LCL cells and the indicated doses of p2 peptide. After 48 hours, the concentration of IFN-γ in the culture supernatant is measured by ELISA. Indicated are the concentration of p2 peptide required to elicit half-maximum release of IFN-γ in the two TCC. (b) Staining of TCC162, TCC51 and the irrelevant TCC with either p2-loaded HLA-DR*1101-Ig or p2-loaded HLA-DR*1101-Bir tetramers. Staining is performed for 2 hours at the indicated temperatures with 10 μg of tetramer per sample. The tetramer staining on CD3^+^CD4^+ ^gated cells is shown. The amount of surface TCR, obtained by staining with anti-CD3 mAb, expressed by the TCC in the different conditions is shown by the mean fluorescent intensity (CD3 mfi).

We finally compared the staining capacity of both soluble HLA-DR*1101 molecules, loaded with different CD4 peptide epitopes. We selected for the staining experiment two p2-specific CD4^+ ^T cell clones displaying low and high affinity for the peptide respectively, as shown in Figure 4a. HLA-DR*1101-Ig or HLA-DR*1101-Bir were loaded with p2 peptide, multimerized and used for the staining at 4°C or 37°C, according to previous data suggesting a significant temperature effect on the interaction between the TCR and the peptide-pulsed MHC-class II tetramers [25,34].

As shown in Figure 4b, HLA-DR*1101-Ig tetramers did never stain either p2-specific T cell clones at any temperature. By contrast, HLA-DR*1101-Bir tetramers stained both low and high affinity p2-specific T cells, although the low affinity T cells clone could only be stained at 37°C, while the high affinity one could be stained at both temperature, though with a much higher fluorescence intensity at 37°C. Collectively these results indicated that HLA-DR*1101-Bir tetramers stain peptide-specific CD4^+ ^T cells, and required an optimal temperature of staining of 37°C. By contrast, HLA-DR*1101-Ig could not stain peptide-specific T cells, despite their capacity to be loaded with peptides.

### Staining with HLA-DR*1101-Bir tetramers depends on the activation state of CD4^+ ^T cells

**Figure 5:**
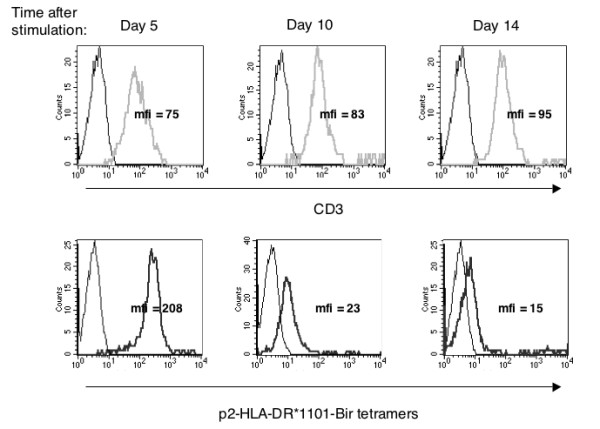
HLA-DR*1101-Bir tetramer staining correlates with the activation state of the target CD4^+ ^T cells. The p2-specific CD4^+ ^TCC 162 was stained with p2-loaded HLA-DR*1101-Bir tetramers at different time after in vitro restimulation with p2 and APCs. The histograms in the upper row display the amount of surface TCR (mfi) expressed by the T cells at the time of tetramer staining, as determined by anti-CD3 staining. The histograms in the lower row show the staining with the p2-loaded HLA-DR*1101-Bir tetramers (mfi) performed at the indicated time after restimulation.

In an independent series of experiments, we noticed that the staining level of CD4^+ ^T cells by peptide-pulsed HLA-DR*1101-Bir molecules was consistently higher when T cells had been recently restimulated with either cognate peptide or polyclonal stimuli in the presence of PBMCs as APCs. This observation prompted a more systematic investigation of the phenomenon. This is in fact a relevant issue when considering the use of HLA-DR*1101 tetramers for *ex vivo *staining, since primary CD4^+ ^T cells might display a whole range of different activation states, which in turn could affect the "detectability" of the whole peptide-specific T cell repertoire by the HLA-DR*1101 tetramers. We therefore compared the capacity of HLA-DR*1101-Bir tetramer to stain the high affinity p2-specific CD4^+ ^T cell clone 162 at different time points from restimulation. As shown in Figure 5, the 162 T cell clone could be stained progressively less by p2-HLADR1101 tetramers moving from day 5 to day 14 post-restimulation, even though the mean fluorescence value of the cell-surface CD3-TCR complex was not substantially modified in the three conditions. This finding confirmed that the activation state of the target CD4^+ ^T cells influences the binding of HLA-DR*1101-Bir tetramers to the TCR.

### HLA-DR*1101-Bir tetramers stain polyclonal CD4^+ ^T cells specific for p2 or Influenza Hemagglutinin HA peptides

**Figure 6:**
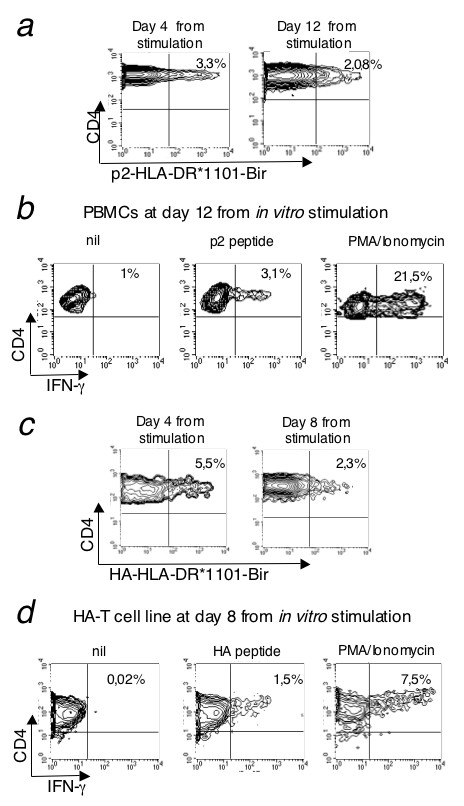
**Peptide-loaded HLADR1101-Bir tetramers stain polyclonal T cells specific for p2 and HA**. *In vitro *expanded T cells were stained with HLA-DR*1101-Bir tetramers loaded with either p2 or HA peptides. (a) Staining of PBMCs from a HLA-DR*1101 healthy donor after one round of *in vitro *stimulation with 5 μM of p2-peptide and 40 u/ml of IL-2. (b) Intracellular production of IFN-γ by T cells contained in the p2-enriched PBMC. T cells were stimulated for 6 hours with p2 in the presence of HLA-DR*1101^+ ^LCL cells at 37°C. Brefeldin A was added after the first hour of stimulation. The cells were then fixed, permeabilised and stained with anti-IFN-γ mAb. Cells unstimulated (nil) and stimulated policlonally with PMA-Ionomycin (PMA/ionomycin) are shown as controls. Numbers in quadrants indicate the percentage of T cells stained with anti-IFN-γ mAb (c) Staining of PBMCs from HLA-DR*1101 healthy donor with HA-loaded HLA-DR*1101-Bir tetramers. T cells were after three rounds of *in vitro *stimulation with 1 μg/ml of HA peptide and 40 u/ml of IL-2. The staining is performed at the indicated days after the third stimulation. Numbers in quadrants indicate the percentage of T cells stained by HLA-DR tetramers. (d) Intracellular production of IFN-γ by CD4^+ ^T cells contained in the HA-specific T cell line at day 8 from the third *in vitro *stimulation. Activation and staining of CD4^+ ^T cells was performed as described in (b).

We next verified the capacity of HLA-DR*1101-Bir tetramers to detect polyclonal antigen-specific CD4^+ ^T cells. Two different helper epitopes from proteins expressed by environmental pathogens were selected: TT-p2 and Influenza Hemagglutinin HA_306–318 _(HA) [35], another well characterised and commonly utilised promiscuous CD4 epitope. PBMCs from healthy donors were stained with p2-pulsed or HA-pulsed HLA-DR*1101-Bir tetramers either *ex vivo *or at different time points after in vitro stimulation with the corresponding peptide. In line with published data [24,26,36], p2- or HA-specific CD4^+ ^T cells could be hardly detected *ex vivo *by HLA-DR*1101-Bir tetramers pulsed with either peptides (data not shown), with frequencies of tetramer positive cells in the range of those obtained with peptide-unloaded (empty) HLA-DR*1101 tetramer (0,05–0,1%). After 4 days of stimulation *in vitro *with the specific peptide, however, a sizeable fraction of CD4^+ ^T cells could be clearly detected with either peptide-pulsed-HLA-DR*1101-Bir tetramer (Figure 6a,c), with a background staining with peptide-unloaded tetramers in the order of 0,05–0,07% in the two T cell cultures (data not shown). The percentage of CD4^+ ^T cells stained with the tetramers paralleled fairly accurately the percentage of cells that were able to produced IFN-γ upon *in vitro *stimulation with the corresponding peptide at the same time point (Figure 6b,d). Again, the percentage of CD4^+ ^T cells stainable with peptide-HLA-DR*1101-Bir tetramers declined with time following the *in vitro *stimulation with the peptide, confirming at the polyclonal level what observed with T cell clones specific for p2, although the decline of staining of HA-specific CD4^+ ^T cells was more rapid than that of p2-specific ones.

### HLA-DR*1101-Bir tetramers stain CD4^+ ^T cells specific for the MAGE-3 tumour antigen derived M3_191–205 _(p39) peptide

**Figure 7:**
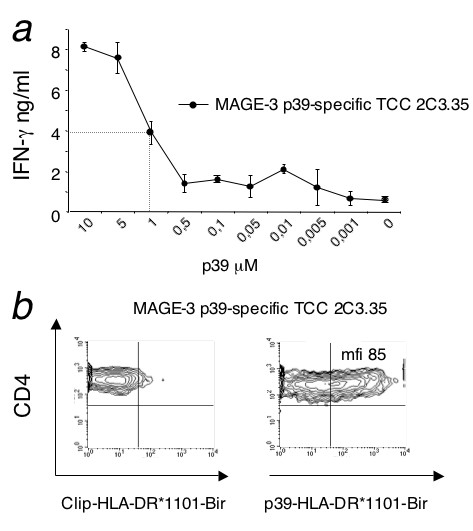
HLA-DR*1101-Bir tetramers stain CD4^+ ^T cells specific for the MAGE-3 tumour antigen derived p39 epitope. Ability of p39-loaded HLA-DR*1101-Bir tetramers to stain a specific CD4^+ ^TCC. (a) Clone M3 2C3.35 was assayed for IFN-γ production to different doses of p39 peptide. 10^4 ^T cells are incubated for 48 hours with 5 × 10^4 ^HLA-DR*1101^+ ^LCL cells and the indicated doses of p39 peptide. IFN-γ in the supernatant is measured by ELISA. (b) Staining of a MAGE-3-specific T cell clone. The cells are stained at day 19 from re-stimulation with 10 μg of either CLIP-loaded or p39-loaded HLA-DR*1101-Bir tetramers for 2 hours at 37°C.

We were also interested in determining whether HLA-DR*1101-Bir tetramers could stain CD4^+ ^T cells specific for the naturally processed, promiscuous p39 peptide derived from the MAGE-3 TAA [37]. As shown in Figure 7, the p39-specific CD4^+ ^T cell clone 2C3.35 could be stained with p39-pulsed HLA-DR*1101-Bir tetramers. The affinity of this CD4^+ ^T cell clone for the p39 peptide, as shown in a dose response experiment of IFN-γ production (Figure 7b), was even lower to that of the low affinity T cell clone 51 for p2 peptide, explaining the low mfi value obtained by staining the 2C3.35 T cell clone with p39-HLA-DR*1101-Bir tetramers. Thus, HLA-DR*1101-Bir tetramers could be pulsed also with a naturally processed promiscuous tumour-derived peptide, and might prove useful in characterising naturally arising or therapeutically induced CD4^+ ^T cells responses in HLA-DR*1101 patients with tumours expressing MAGE-3.

## Discussion

We have shown the generation of a versatile HLA-DR*1101 tetramer. A single soluble recombinant protein backbone can bind at least three different promiscuous peptide epitopes, derived from environmental pathogens or tumour associated antigens, by a simple incubation step. This feature will greatly facilitate the use of this reagent for the study of CD4^+ ^T cells responses specific for disparate antigens. The usefulness of this approach is evident also from the studies performed with HLA-DR*0401 and HLA-DR*0404 tetramers [23-26], in which the same protein backbone was loaded with either viral or self-derived peptide. The only HLA-DR*1101 tetramers described so far are produced in *E coli *with a covalently linked HCV-derived peptide [38] and, although functional, are limited in their use to the single peptide specificity. Therefore, the production of versatile HLA-DR*1101 tetramers is extremely valuable considering the frequency of expression of this allele in the Caucasian population.

It is interesting to note that the same extracellular portion of the HLA-DR allele behaves differently in terms of stability when engineered with different C-terminal moieties. The presence of an Ig constant domain at the C-terminus seems in fact to destabilise the "empty" HLA-DR*1101 soluble recombinant molecule significantly more than the presence of the Bir sequence. The most straightforward explanation for this phenomenon holds that the extended intermolecular interactions introduced by the pairing of two Ig Fcs may disturb the native conformation of the chimeric HLA molecules. In fact our results show that the addition of a short Bir sequence at the C-terminus does not impact on the overall molecular architecture of this human HLA-DR allele. Evidence for a conformational alteration in the HLA-DR*1101-Ig molecule stems from the immuno-precipitation experiments with the anti-HLA-DRα chain-specific monoclonal antibody L243, which recognizes a conformational epitope that is present only when the HLA-DRα and β chain are correctly paired [30,31]. L243 immunoprecipitates very inefficiently HLA-DR*1101-Ig molecules, whereas it efficiently binds HLA-DR*1101-Bir, suggesting differences in the structures of the two proteins or a masked epitope in the former. Several mouse MHC class II alleles have nevertheless been successfully produced as chimeric MHC-Ig fusion proteins, with or without a linked peptide, and it is possible that HLA-DR alleles other than DR1101 might be produced in this chimeric form.

It is clear from our study that the affinity of the TCR expressed by the CD4^+ ^T cells for the cognate peptide-HLA-DR*1101 complex will dictate the success of detection of specific primary lymphocyte population by the fluorescent tetramer. Moreover, we document that the activation state of the target CD4^+ ^T cells affects the binding of peptide-HLA-DR tetramers to the cognate TCR, suggesting that biochemical pathways linked to CD4^+ ^T cell activation modify the avidity of the TCR for the HLA-DR tetramer. The fact that the binding of the TCR by the cognate peptide-HLA-DR*1101 tetramers requires an active metabolism and intact membrane trafficking is suggested further by the 37°C temperature requirement for optimal CD4^+ ^T cell staining. Collectively, these observations might help to explain why the detection *ex-vivo *of primary CD4^+ ^T cells by MHC class II tetramers seems more difficult than the detection of specific CD8^+ ^T cells by MHC class I tetramers. Further study are needed to determine whether modifying rationally the structure of the helper peptide epitope to increase its affinity for the MHC class II allele and/or the cognate TCR, and manipulating the T cell activation pathways to increase the avidity of the TCR for HLA-DR tetramers, my lead to improved *ex-vivo *detection of specific CD4^+ ^T cells.

## Conclusion

In conclusion, we report here the successful engineering of stable empty HLA-DR*1101-Bir tetramers. These molecules are novel powerful reagents for the study of CD4^+ ^T cell responses towards diverse peptide antigens. Our comparative study on the production of two different "empty" HLA-DR*1101 constructs, together with the evidences present in the literature, suggests that every MHC class II isotype, and even allele, might display distinct biochemical characteristics, requiring the empiric definition of the optimal strategy for the production as functional soluble recombinant molecules.

## Methods

### Construction of soluble recombinant HLA-DR*1101-Ig and HLA-DR*1101-Bir molecules for insect cells expression

All the DR α and β constructs were cloned in the pMT/Bip/V5-His vector (Invitrogen, Groningen, the Nederlands) in frame with the *Drosophila *BiP secretion signal, under the control of the Drosophila metallothionein promoter.

The extracellular region of the HLA-DRα chain, deprived of the leader sequence, was cloned by RT-PCR using the following primers: oligo-1-Up DRα (BglII) 5'GGGAGATCTATCAAAGAAGAACATGTGATCATCCAG3', oligo-2-Dw DRα (BamHI) 5'GGATCCTCCACCTCCGTTCTCTGTAGTCTCTGGG3'. The PCR product was cloned into the PCR2.1 vector (Invitrogen) and sequenced.

To generate the HLA-DRα-Bir chain, a cassette containing the sequence for the Acidic Leucine Zipper-Bir A target peptide was generated by fusion PCR with the following primers, as illustrated in Figure 1:

Oligo-3-Up AZ (BamHI) 5'GGAGGATCCACTACAGCTCCATCAGCTCAG3', Oligo-4-Dw AZ (Bir) 5'ACCACCACCGTCCCCACCCTGAGCCAGTTC3', Oligo-5-Up Bir (AZ) 5'GGTGGGAGCGGTGGTGGTCTGAACGATATTTTTG3' and Oligo-6-Dw Bir (NotI) 5'CTAGCGGCCGCTATTCATGCCATTCGAT3'. The PCR product was cloned into the PCR2.1 vector and sequenced, then subcloned in frame with the HLA-DRα fragment separated by a five-aminoacid linker (GGGGS). The resulting sequence encoding the HLA-DRα-AZ-Bir fusion protein was excised with BglII and NotI restriction enzymes, and cloned into the Drosophila melanogaster expression vector pMT/Bip/V5/His.

To generate the HLA-DRα-Ig chain, a cassette containing the sequence for the Acidic Leucine Zipper-hIgG1 constant region was generated by fusion PCR as illustrated in Figure 1, using the following primers:

oligo-3-upAZ(BamHI) described above, oligo-9-Dw AZ (Ig) AGATTTGGGCTCAGATGCTGCCTGAGCCAGTTC, oligo-10-Up Ig (AZ) GCAGCATCTGACGCCCAAARCTTGTGACAAAACTCAC and oligo-11-Dw IgG1 (NotI) 5'TGGCGGCCGCCGCACTCATTTACCCGGAGA. The PCR product was cloned into the PCR2.1 vector, sequenced and then subcloned in frame with the HLA-DR*1101α chain fragment. The resulting sequence encoding for the HLA-DR*1101α-AZ-IgG1 fusion protein was excised with BglII and NotI, and cloned in the Drosophila melanogaster expression vector pMT/Bip/V5/His.

The HLA-DR*1101β chain, shared by both type of soluble recombinant HLA-DR*1101 molecules, was generated as follows. The extracellular region of the HLA-DR*1101β, chain deprived of its leader sequence, was cloned by RT-PCR using the following primers:

oligo-7-Up DRβ (BglII) 5'GGGAGATCTGGGGACACCAGACCACGTTTC3', oligo-8-Dw DRβ (BamHI) 5'GTGGATCCTCCACCTCCCTTGCTCTGTGCAGATTC3'. The PCR product was cloned into the PCR2.1 vector and sequenced. The HLA-DR*1101β cDNA was subcloned in frame with a Basic Leucine Zipper-His tag cassette derived from the pCO268 plasmid spaced by a five-aminoacid linker (GGGGS) [21]. The resulting coding sequence was then excised with BglII and SalI restriction digestion, and cloned into the Drosophila melanogaster expression vector pMT/Bip/V5/His, digested with BglII and XhoI.

### Expression of HLA-DR*1101 in Drosophila cells

S2 cells were grown in SFX medium (Hy-clone, South Logan, UT, USA) supplemented with 10% heat inactivated foetal calf serum (Euroclone, Milano, Italy), 50 u/ml penicillin, 25 μg/ml streptomycin, 25 μg/ml Kanamycin (Gibco) and 1 μg/ml Amphotericin B (Gibco, Paisley, Scotland, UK) at 27°C to a density of 2–4 × 10^6 ^cells/ml. The α- and β-chain expressing vectors (15 μg each) were co-transfected with 0,5 μg of the selection plasmid pCoHYGRO using a calcium phosphate transfection kit (Invitrogen). Three days after the transfection, selection medium containing 300 μg/ml of Hygromicin B (Roche, Indianapolis, IN, USA) was added to cells. Transfected cells were cultured in SFX medium supplemented with 2% of foetal calf serum and 300 μg/ml of Hygromicin B. Cells were kept in the exponential growth phase (7–20 × 10^6 ^cells/ml) and the protein production was induced by the addition of 1 mM of CuSO4 when the cells were at a density of 10^7^/ml. Protein production was monitored by dot blot analysis with either mouse anti-human HLA-DR α monoclonal antibody L243 (ATCC, Manassas, VA, USA) or rabbit anti-His tag polyclonal antibody His-probe (Santa Cruz, Santa Cruz, CA, USA), followed by an HRP-labelled goat anti mouse or anti rabbit antibody (Southern Biothecnology, Birmingham, AL, USA). The assay was developed with ECL (Amersham, Uppsala, Sweden). To improve the production of recombinant proteins, transfected cells were cloned by limiting dilution. S2 cells expressing the desired HLA-DR*1101 molecule were diluted to 2.5 cell/ml in a suspension of irradiated (8000 rad) S2 wild type cells at the concentration of 3 × 10^5 ^cells/ml in medium+Hygromicin B, and plated in flat bottomed 96 w plates. The best producer clones were selected by dot blot analysis on the culture supernatant as described above, upon induction with 1 mM of CuSO4 of the same number of cells, and used for routine protein production.

### Purification of soluble HLA-DR*1101 molecules

Both HLA-DR*1101-Ig and HLA-DR*1101-Bir molecules were purified from the S2 cells supernatant by immunoaffinity chromatography using ProtA-Sepharose (Amersham Bioscience) and HLA-DRα-specific L243 mAb-Sepharose, respectively. The HLA-DR*1101-Ig molecules were stored at -80°C at a concentration of 1 mg/ml in PBS buffer. HLA-DR*1101-Bir molecules were dialyzed against 10 mM Tris buffer, pH 8.0, and concentrated up to 3 mg/ml on Vivaspin2 concentrator (Vivascience, Hannover, Germany). The protein was biotinylated using the BirA enzyme according to the manufacturer's instructions (Avidity, Denver, CO, USA), then was diluted to 1 mg/ml and stored at -80°C. The formation of molecular aggregates was assessed by size exclusion chromatography on a Superdex 200 HR 10/30 column using a ÅKTA FPLC chromatography system (Amersham Bioscience). The calibration of the Superdex 200 column was performed with the Gel Filtration Calibration Kit high molecular weights (Amersham Bioscience), following the manufacturer indications. Partition coefficients were calculated using the relation Kd = (Ve-Vo)/(Vc-Vo), where Ve, Vc and Vo are the elution, column and void volumes, respectively.

### Peptide loading into soluble HLA-DR*1101 molecules

Both HLA-DR*1101-Ig and HLA-DR*1101-Bir were loaded with synthetic peptides by incubation at 37°C for 72 hours with 50 fold molar excess of either Tetanus Toxoid peptide residues 829–844 (p2), Hemagglutinin peptide residues 306–318 (HA) or MAGE-3 tumour associated antigen peptide residues 191–205 (p39) in 10 mM Tris, 50 mM Glycine pH 5.0 with 0.2% of n-octyl-β-D-glucopyranoside (Sigma-Aldrich, St. Louis, MO, USA).

### Tetramerization of soluble HLA-DR*1101 molecules and staining of specific T cells

Tetramerization of HLA-DR*1101-Ig and HLA-DR*1101-Bir were achieved by adding FITC-labelled protein A (Molecular Probes, Leiden, The Netherlands) at 5:1 molar ratio, or PE-labelled streptavidin (Molecular Probes) at 5:1 molar ratio, respectively. Tetramer staining was performed for 2 hours at 37°C in complete medium. The cells are then washed twice, transferred on ice and stained for the other surface markers. Immediately before the analysis TOPRO-3 (Molecular Probes) is added following the manufacturer's instructions to exclude dead cells. The analysis is performed on CD3^+^CD4^+ ^viable cells.

### Generation of specific CD4^+ ^T cell lines and clones

20 million of freshly purified PBMCs from a HLA-DR*1101/1104 healthy donor were cultured for 7 days in RPMI 1640 (Gibco) supplemented with 10% of human serum, 2 mM Glutamax I (Gibco), 100 u/ml penicillin and 50 μg/ml streptomycin (Gibco), in the presence of the specific peptide at a concentration ranging from 1 to 10 μg/ml. After seven days, blasts were separated from small T cells on a 30–70% discontinuous Percoll gradient [39], and put in culture in complete medium with 10–20 u/ml of human recombinant IL-2 (Roche). Peptide specific T cells were kept in culture by weekly re-stimulation with the same amount of peptide and irradiated (4000 rad) autologous PBMCs as antigen presenting cells. Peptide-specific T cell clones were generated by limiting dilution of Percoll-purified T cell blasts blasts as follows. Blasts were diluted at 25 cells/ml in a suspension containing 0.5 × 10^6 ^cells/ml of a mixture of two different allogeneic PBMCs supplemented with 1 μg/ml of PHA-L (Roche) and 200 u/ml of human recombinant IL-2 (Roche). The cloning mix is dispensed in 25 μl in flat-bottomed Terasaki plates and incubated at 37°C for 10–20 days. Growing cells are then tested for the recognition of peptide pulsed HLA matched EBV cell lines.

### T cells functional assays

To test IFN-γ production, 10^4 ^T cells were plated in U bottom 96 well plates in RPMI 1640 supplemented with 10% of human serum, 2 mM Glutamax I, 100 u/ml penicillin, 50 μg/ml streptomycin, together with 5 × 10^4 ^irradiated (6000 rad) HLA-DR*1101 LCL cells as antigen presenting cells, and the specific peptide at the concentrations detailed in the figure legends. IFN-γ production in the supernatant was measured by ELISA after 48 hours of incubation (IFN-γ detection kit-Endogen).

For the intracellular analysis of IFN-γ production, T cells were stimulated for 6 hours with HLA-DR*1101 LCL of at 1:5 ratio in three conditions: *i*. without peptide; *ii*. with 5 μg/ml of peptide; and *iii*. with 50 ng/ml of PMA (Sigma) plus 500 ng/ml of Ionomycin (Sigma). After the first hour of stimulation, Brefeldin A was added at the final concentration of 10 μg/ml. At the end of the stimulation, the cells were harvested, fixed with PFA1%, permeabilized with 0,5% saponin and stained for intracellular cytokine expression.

For the T cell activation with peptide-loaded soluble recombinant HLA-DR molecules, 1 μg of peptide loaded HLA-DR*1101-Ig or HLA-DR11-Bir molecules in 50 μl of PBS were incubated in U bottomed 96 well plates for 3 hours at 37°C. After washing, 2 × 10^4 ^specific T cells were added to the wells and incubated for 48 hours at 37°C in the presence of 1 ng/ml of PMA. Cells incubated either with PMA alone or in the presence of 1 μg of plate coated TR66 anti-CD3 mAb are used as negative and positive control, respectively. IFN-γ production in the supernatant is measured by ELISA (Endogen, Woburn, MA, USA) after 48 hours of culture.

### Antibodies and flow cytometry

The following antibodies have been used: FITC- and APC-labelled mouse anti human CD3 (Becton Dickinson, San Jose, CA, USA), Quantum Red-conjugated mouse anti-human CD4 (SIGMA), PE-labelled rat anti-human IFN-γ (Becton Dickinson). Data were collected using a Becton Dickinson FACScalibur flow cytometer and analysed using the Cell Quest software (Becton Dickinson)

## Authors' contributions

M.M, P.D., G.C designed experiments. M.M, V.C., C.M., E.D. produced and characterised the tetramers. B.G., M.D. contributed to the purification of the HLA-DR monomers. N.G., M.P.P contributed reagents and expertises. M.M, V.C., P.D., G.C. analysed data. M.M, P.D., G.C. wrote the paper. All authors read and approved the final manuscript.
